# Transcriptome of the Plant Virus Vector *Graminella nigrifrons,* and the Molecular Interactions of *Maize fine streak rhabdovirus* Transmission

**DOI:** 10.1371/journal.pone.0040613

**Published:** 2012-07-12

**Authors:** Yuting Chen, Bryan J. Cassone, Xiaodong Bai, Margaret G. Redinbaugh, Andrew P. Michel

**Affiliations:** 1 Department of Entomology, Ohio Agricultural Research and Development Center (OARDC), The Ohio State University, Wooster, Ohio, United States of America; 2 United States Department of Agriculture-Agricultural Resarch Service, Corn and Soybean Research and Department of Plant Pathology, Ohio Agricultural Research and Development Center (OARDC), The Ohio State University, Wooster, Ohio, United States of America; U. Kentucky, United States of America

## Abstract

**Background:**

Leafhoppers (Hemiptera: Cicadellidae) are plant-phloem feeders that are known for their ability to vector plant pathogens. The black-faced leafhopper (*Graminella nigrifrons*) has been identified as the only known vector for the *Maize fine streak virus* (MFSV), an emerging plant pathogen in the *Rhabdoviridae*. Within *G. nigrifrons* populations, individuals can be experimentally separated into three classes based on their capacity for viral transmission: transmitters, acquirers and non-acquirers. Understanding the molecular interactions between vector and virus can reveal important insights in virus immune defense and vector transmission.

**Results:**

RNA sequencing (RNA-Seq) was performed to characterize the transcriptome of *G. nigrifrons.* A total of 38,240 ESTs of a minimum 100 bp were generated from two separate cDNA libraries consisting of virus transmitters and acquirers. More than 60% of known *D. melanogaster, A. gambiae, T. castaneum* immune response genes mapped to our *G. nigrifrons* EST database. Real time quantitative PCR (RT-qPCR) showed significant down-regulation of three genes for peptidoglycan recognition proteins (*PGRP – SB1*, *SD*, and *LC*) in *G. nigrifrons* transmitters versus control leafhoppers.

**Conclusions:**

Our study is the first to characterize the transcriptome of a leafhopper vector species. Significant sequence similarity in immune defense genes existed between *G. nigrifrons* and other well characterized insects. The down-regulation of *PGRP*s in MFSV transmitters suggested a possible role in rhabdovirus transmission. The results provide a framework for future studies aimed at elucidating the molecular mechanisms of plant virus vector competence.

## Introduction

Hemipteran insects such as aphids, whiteflies, planthoppers and leafhoppers are arguably the most important vectors of plant-infecting viruses. These insects have specialized mouthparts suitable for tissue specific feeding (often the phloem), and wide host ranges that provide ample opportunity for virus transmission [1], [2]. Most insect vectors of plant viruses have significant associations with humans and agroecosystems. Recent rapid changes in these environments have increased crop exposure to viruses and vectors or altered evolutionary, ecological or genetic interactions leading to enhanced transmission [3]. A lack of understanding these factors, including the molecular mechanisms of virus transmission by vectors, reduces our ability to assess and manage risks posed by plant virus vectors, particularly for emerging diseases whose full impacts are not yet realized.

*Graminella nigrifrons* is one of the most common and abundant leafhoppers in the eastern half of the U.S., presently found in 35 states from southern Maine to Florida [4]. It has a wide plant host range, including oats (*Avena sativa* L.), maize (*Zea mays* L.), perennial rye grass (*Lolium perenne* L.) and johnsongrass (*Sorghum halepense* L.) [5]. *G. nigrifrons* is a natural and experimental vector of several pathogens (e.g. maize bushy stunt phytoplasma and corn stunt spiroplasma) as well as several types of viruses, including rhabdoviruses [6]–[10].

*G. nigrifrons* has recently been identified as a vector of the emerging Rhabdovirus *Maize fine streak virus* (MFSV) [7], [11], which is a newly described member of *Nucleorhabdovirus*, first detected in Georgia, US in 1999 [7]. In maize, this emerging virus causes symptoms of dwarfing and fine chlorotic steaks along intermediate and small veins. Consistent with most rhabdoviruses, MFSV has high vector species specificity as *G. nigrifrons* is the only reported insect vector [7], [11], [12]. *G. nigrifrons* transmits MFSV in a persistent propagative manner, but, vector competence for MFSV is not consistent within *G. nigrifrons* populations. Individual insects can be categorized into three groups according to their acquisition or transmission capabilities after exposure to MFSV infected plants. ‘Transmitters’ acquire and transmit virus (i.e., they are vectors); ‘acquirers’ acquire, but cannot transmit virus; and non-acquirers do not acquire virus after feeding on infected plants [7], [11]. Mechanisms of these differences are unknown, but it is hypothesized that barriers to virus replication and movement within the insect might be the key factors limiting vector competence among *G. nigrifrons* individuals [13], [14]. While biological parameters associated with MFSV and other pathogen transmission by *G. nigrirons* are defined, the absence of molecular resources for this vector prevents further investigations into insect vector competence, the molecular basis of rhabodvirus transmission, and comparative vector genomics. While a few molecular investigations exist for planthoppers [15], [16], data for leafhoppers is virtually non-existent.

Rhabdoviruses can infect both plants and animals (including mammals) and consist of a nucleocapsid containing a negative single-stranded RNA genome encapsidated into a lipid bilayer membrane. There are seven recognized *Rhabdoviridae* genera, but only viruses within the *Nucleorhabdovirus* and *Cytorhabdovirus* infect plants [17], [18]. In contrast to most plant viruses, the rhabdoviruses also infect their insect vectors, they must, therefore, replicate within the insect and cross several molecular and physical barriers prior to being transmitted to additional host plant [14]. These barriers may include digestive enzymes, peritrophic lining, improper epithelial cell receptors for virus attachment, programmed cell death, virus induced RNA interference (RNAi) or an active insect immune response [14], [19]. A midgut barrier to virus infection of poor vectors has been defined for some rhabdoviruses, but other barriers are not as well understood [14], [18].

In other systems, the impact of several insect responses on vector competence has begun to be defined, including insect innate immunity, a highly conserved, essential defense against microbial infection, such as bacteria, fungi, nematodes and viruses [20]. Non-self molecules are recognized and subsequently trigger cell-mediated phagocytosis, encapsulation and/or humoral responses. The humoral response is activated by the binding of peptidoglycan recognition proteins (PGRPs) to pathogen associated molecular patterns (PAMPs), which then regulate antimicrobial peptide (AMPs) production through Toll or immune deficiency (IMD) signaling cascades [21]. Two classes of PGRPs are described: the short PGRPs (PGRP-S) are small extracellular proteins without transmembrane domains present in the hemolymph and cuticle; and, long PGRPs (PGRP-L) are larger proteins that encode transmembrane domains [22]. The responses of insect PGRPs to bacterial and fungal infection have been well described, and several recent studies revealed the involvement of the Toll pathway and PGRPs in *D. melanogaster* defense against viral infection [19], [23]. The number and the type of PGRPs vary among insects. For example, *D. melanogaster* has 12 PGRP genes [22] and *Anopheles gambiae* has 7 [24], but the Hemipteran pea aphid *Acyrthosiphon pisum* lacks PGRPs due to the co-evolution with its endosymbionts [25]. The role of PGRPs in leafhoppers and planthoppers, the primary vectors of plant viruses, is not well understood.

RNA interference (RNAi) is another key pathway protecting eukaryotic organisms from virus infection [26], [27]. Genes, proteins and pathways important for RNAi have been characterized in *D. melanogaster*, but genes involved in these pathways have just begun to be described in insect vectors of plant viruses including *Peregrinus maidis* [28], and *A. pisum* [29]. No information about RNAi pathway genes is available for leafhoppers.

To expand leafhopper molecular resources, we constructed cDNA libraries using RNA from *G. nigrifrons* transmitters and acquirers, and sequenced the transcripts using RNA-seq. The resulting EST database provides the first transcriptome information on a leafhopper, and contains genes potentially involved in anti-pathogen defense. In addition, we used RT-qPCR to examine the expression of seven genes putatively involved in insect immune response for leafhoppers known to transmit MFSV and leafhoppers not previously exposed to MFSV.

## Methods

### Insect rearing and virus maintenance

*G. nigrifrons* was maintained on maize (*Zea mays* L.) hybrid Early Sunglow in growth chambers, and MFSV was maintained by serial transfer to maize at OARDC as previously described [11]. For virus maintenance, 300 *G. nigrifrons* individuals were reared on MFSV infected maize for three weeks then transferred to healthy maize seedlings. Inoculated plants with MFSV symptoms were subsequently used as source plants.

### Differentiating non-acquirers, acquirers, and transmitters

Two hundred *G. nigrifrons* adults (100 males and 100 females) were collected, placed in a single cage, and allowed to mate and oviposit on MFSV infected maize seedlings for two days. After 14 days, the (F_1_) nymphs were observed, and this was denoted as day 0. The F_1_ nymphs were reared on MFSV infected maize seedlings for 21 days, then F_1_ adults were individually transferred to 4-day old healthy maize plants within a tube. After one week, F_1_ leafhoppers were collected, labeled according to the maize test plant, and stored at −80°C. Plants were moved to a growth chamber for three weeks for MFSV symptom development [11]. An insect was designated as a transmitter if MFSV symptoms developed on the test plant it fed on. For non-transmitter insects, RT-PCR was performed on individuals using the Access RT-PCR System (Promega, Madison, WI) following the manufacturer's protocol. Primer pairs 361F (5′-GTGCAGAATTGCCCTATCC-3′)/917R (5′-TCGAGGCAATTCCTGTATC-3′) and 5335F (5′-CTCCCATTATCATAGATAAAG-3′)/6360R (5′-TATATGCAATTCTGATTCCTC-3′) were used to amplify a 1120 bp and 1030 bp fragments of the viral N and G genes, respectively, based on MFSV genome sequence [12]. Reverse transcription was performed at 45°C for 45 min and followed by 2 min at 94°C. PCR included 40 cycles of 94°C for 30 s, annealing at 54°C (N gene) or 52°C (G gene) for 30 s, and extension at 68°C for 120 s. An insect was designated as an ‘acquirer’ if RT-PCR indicated the presence of MFSV, but no transmission of the virus to the test plant. ‘Non-acquirers’ were those insects for which MFSV was not detected by RT-PCR, and no symptoms developed on test plants.

### RNA isolation and cDNA library construction

Total RNA from individual *G. nigrifrons* transmitters and acquirers was isolated with Trizol (Invitrogen, Grand Island, NY) following the manufacturer's protocol. The concentration and the quality of RNA were analyzed using a Nanodrop 2000c spectrophotometer and Agilent 2100 Bioanalyzer. RNA (10 µg) was pooled from eight individuals from each class and used to construct two cDNA libraries following the mRNA sequencing sample preparation guide (Illumina, San Diego, CA). Paired-end DNA sequencing was done in two lanes (one per library) on an Illumina GA-II following manufacturer's protocol.

### Sequence assembly and annotation

The 76-bp paired-end Illumina reads from the acquirer (32,548,016 reads) and transmitter (30,541,892 reads) libraries were combined for *de novo* assembly. Low-quality (≥80% of the read with the Phred score of less than 20) and low complexity (>80% of the read with single-nucleotide, di-nucleotide, or tri-nucleotide repeats) were removed. The processed reads were then assembled using a combination of the Velvet (ver. 1.2.01) [30] and Oases (ver. 0.2.06; http://www.ebi.ac.uk/~zerbino/oases/) programs with the k-mer lengths of 41, 43, 45, 47, 49, 51, 53, and 55. The resulting assembled sequences and singletons were combined, processed to remove duplicates using a custom Perl Script [31], and further assembled after examining the overlapped regions identified by Vmatch (ver. z.z) [32]. Contigs were then further assembled using Phrap program (version: 0.020425.c) [33] to obtain the final transcriptome of sequences >100 bp. Sequences have been submitted to the short read archive at NCBI GenBank under accession number SRP013390.3.

Functional annotation of the *G. nigrifrons* transcriptome was performed by searching for analogous sequences in the Swiss-Prot database (http://www.ebi.ac.uk/uniprot) using an E-value cut-off of 10^−4^. Hierarchical functional categorization on gene ontologies (GO terms) was done using BLAST2GO (http://www.blast2go.de) [34]. BLAST2GO was also used to identify genes represented in Kyoto Encyclopedia of Genes and Genomes (KEGG) pathways (http://www.kegg.com/kegg/kegg1a.html).

### Comparison of *G. nigrifrons* and other invertebrate transcriptomes and immune response genes

Each of the 32,480 *G. nigrifrons* ESTs was subjected to pair-wise comparison to EST databases of eight invertebrate species using desktop downloaded tBLASTx software and a 10^−10^ E-value threshold. Seven invertebrate databases were constructed by retrieving cDNA sequences of characterized genomes from NCBI (ftp://ftp.ncbi.nih.gov) or Ensembl (ftp://ftp.ensembl.org/): *Acyrthosiphon pisum* (pea aphid, order Hemiptera, 37,994 sequences), *Apis mellifera* (honey bee, order Hymenoptera, 18,542 sequences), *Nasonia vitripennis* (parasitic wasp, order Hymenoptera, 27,287 sequences), *Tribolium castaneum* (red flour beetle, order Coleoptera, 14,366 sequences), *Anopheles gambiae* (malaria mosquito, order Diptera, 14,974 sequences), *Drosophila. melanogaster* (fruit fly, order Diptera, 19,233 sequences), and *Caenorhabditis elegans* (soil nematode, order Rhabditida, 32,201 sequences). A NCBI transcriptome database was also constructed for *P. maidis* (maize planthopper, order Hemiptera), which contained 10,636 sequences derived from the gut [28]. Immunity gene homologs from *G. nigrifrons* EST database were identified by similarity to annotated immunity genes from an insect immunity gene databases constructed by Whitfield et al. [28] that contained over 300 immunity genes derived from *D. melanogaster* (356 genes) [35], *A. gambiae* (302 genes) [24], and *T. castaneum* (245 genes) [36] using tBLASTx with an E-value cut-off of 10^−10^.

### Validation of reference genes for gene expression studies

Six candidate housekeeping genes were selected: *alpha tubulin* (*α -TUB*) (GnigEST017131), *elongation factor 1-alpha* (*EF-1α*) (GnigEST008963), *glyceraldehyde-3-phosphate dehydrogenase* (*GAPDH*) (GnigEST000002), *succinate dehydrogenase* (*SDHA*) (GnigEST008732), *ribosomal protein L3* (*RPL3*) (GnigEST000330), *ribosomal protein S13* (*RPS13*) (GnigEST004586). Six treatments were used including MFSV-transmitters, non-acquirers and leafhoppers raised on healthy plants, separated in groups of males and females. Total RNA was extracted from individual *G. nigrifrons* adults using Trizol (Invitrogen). DNA was removed from the RNA samples with the TURBO DNA-free kit (Ambion, Grand Island, NY). The concentration and quality of the RNA were verified as outlined above. Total RNA (1μg) from individual insects was used for cDNA synthesis with the SuperScript III First-Strand Synthesis System (Invitrogen, Grand Island, NY). The cDNA was used as template for RT-qPCR using the iQ SYBR Green Mix (Bio-Rad, Hercules, CA), following the manufacturer's recommendations and primers designed using Beacon Designer 7.0 (BioRad, Hercules, CA). PCR was performed with 5µl of SYBR Green Mix, 2 µl of cDNA template (20ng/µl) and 5 pmole of each primer. Target genes were amplified at 95°C for 3 min followed by 40 cycles of 95°C for 10 s and 60°C for 30 s. Nuclease-free water was used for negative control reactions. PCR efficiency (*E*) was calculated by the equation *E* = 10^[−1/slope]^ [37]. A standard curve was constructed using serial dilutions of pooled individual cDNAs and used to determine the relative expression values for these six reference genes (User Bulletin #2: ABI Prism 7700 Sequence Detection System vide supra) (http://www3.appliedbiosystems.com/). The software geNorm was used to determine the most stable reference gene among the six tested [38].

Expression of seven genes with known function in insect innate immunity was evaluated in transmitters and leafhoppers raised on healthy plants (i.e. never exposed to MFSV infected plants, used as control) using RT-qPCR: *acetylcholine receptor subunit alpha-L1* (*AChR*) (GnigEST002867), *autophagy protein 5* (*ATG 5*) (GnigEST012478), *defensin* (GnigEST011432), *peptidoglycan recognition protein SB1* (*PGRP-SB1*) (GnigEST015027), *PGRP-SD* (GnigEST009213), *PGRP-LC* (GnigEST006324), *tripeptidyl peptidase II* (*TPP II*) (GnigEST021246). RNA isolation and RT-qPCR protocols were carried out as outlined above with primers listed in Table S1. The expression profiles of the seven genes were normalized to the internal control *RPS13*. Nuclease-free water was used as the negative control reactions. For each treatment, three biological replicates composed of RNA isolated from 10 adult leafhoppers were analyzed. The relative accumulation of transcripts in MFSV transmitters and control leafhoppers were determined using the comparative C*_T_* method [39]. A T-test was used to compare mean 2^−ΔCt^ values.

## Results and Discussion

### Sequence assembly and annotation

A total of 38,240 good quality ESTs of a minimum 100 bp were generated for the *G. nigrifrons* transcriptome. Approximately 34% of these transcripts (n = 13,036) mapped to the Swiss-Prot database (E<10^−4^) based on deduced amino acid similarity. Of these, 177 homologs were of prokaryotic origin and therefore removed from further analysis. On a more conserved level (E-value <10^−180^), 1,070 *G. nigrifrons* ESTs have high similarity with genes in the Swiss-Prot database (listed in Table S2). Approximately one-third of these highly conserved genes (n = 355) had highest similarity to a *D. melanogaster* gene. To guide biological interpretation, we used BLAST2GO to examine the associations between gene (GO) and enzyme (EC) ontologies assigned to the *G. nigrifrons* ESTs. We identified 4,488 ESTs (12%) that mapped to GO or EC terms (Figure S1). Of these, 2,634 *G. nigrifrons* ESTs were classified as having function in cellular processes, 2,592 as having binding function, and 2604 as cell parts. KEGG-based pathway analysis using BLAST2GO from ortholog cellular pathways indicated that 882 ESTs could be putatively assigned to one or more of 126 KEGG pathways (Table S3). Thus, our initial functional analysis of the *G. nigrifrons* transcriptome indicates significant similarities with those of previously sequenced organisms, particularly *D. melanogaster*. However, fewer than half of the *G. nigrifrons* ESTs had significant similarity to genes in SWISS-PROT database, and less than 10% could be classified by GO term or KEGG pathway.

### Comparison of *G. nigrifrons* and other invertebrate ESTs

Pair-wise comparison of the *G. nigrifrons* ESTs with cDNA databases for seven well-characterized invertebrate genomes and one insect dbEST collection indicated that ca. 37% (n = 14,259) of the *G. nigrifrons* ESTs had a significant match to at least one of the insect databases (E<10^−10^). When significant *G. nigrifrons* matches to the same predicted or known ortholog protein sequence in different species were collapsed into a single observation, there were 9,635 transcripts with at least one hit to an invertebrate database (∼25% of the total ESTs). Partitioning of the significant matches among the eight invertebrate databases indicated *D. melanogaster* (n = 7,303) and *T. castaneum* (n = 7,028) had the greatest number of matches, whereas the most distantly related species, *C. elegans*, had the fewest (n = 4,777). The number of matches ranged between 6,309 and 6,907 for the remaining five invertebrate comparisons. Differences in the number of matches may be due to genomic evolution, or may more likely reflect different stages of transcriptomic characterization and curation.

**Figure 1:**
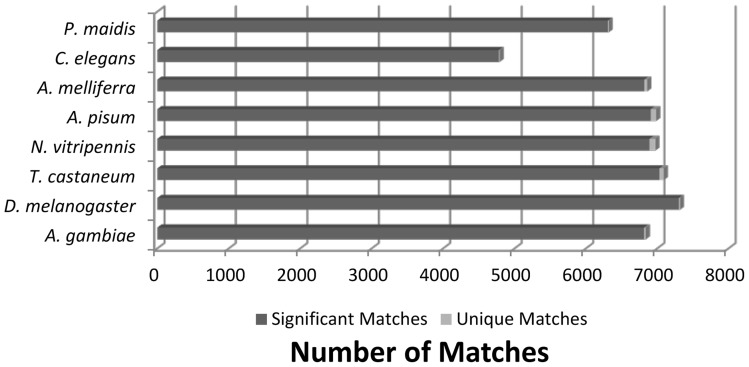
Invertebrate comparative genomics. The number of significant ortholog matches (E-value <10^−10^) as well as the number of unique invertebrate transcripts for all eight pair-wise invertebrate comparisons with *Graminella nigrifrons.* These invertebrates are *Acyrthosiphon pisum* (pea aphid, order Hemiptera), *Apis mellifera* (honey bee, order Hymenoptera), *Nasonia vitripennis* (parasitic wasp, order Hymenoptera), *Tribolium castaneum* (red flour beetle, order Coleoptera), *Anopheles gambiae* (malaria mosquito, order Diptera), *Drosophila. melanogaster* (fruit fly, order Diptera), and *Caenorhabditis elegans* (soil nematode, order Rhabditida).

For the putative or known ortholog transcripts, the number of *G. nigrifrons* ESTs that match a sequence in only one of the eight comparison invertebrate species was determined. The number of significant ortholog matches as well as the number of transcripts exclusive to one invertebrate for all eight pair-wise comparisons are shown in figure 1. Roughly half of the unique matches exclusive to one invertebrate (161 of 332) were attributed to *N. vitripennis* or the hemipteran *A. pisum.* The relatively low percentage (3%) of unique matches to the most closely related species in the comparisons, the corn planthopper *P. maidis*, is likely due to the small number of *P. maidis* ESTs (10,636) compared to the transcriptomes of the other seven query species.

### Immune response ESTs in *G. nigrifrons*

The transcriptional responses of pathogen infection in insect human disease vectors are well described [40]–[46]. These studies suggest that innate immunity genes can have different functions in different genetic backgrounds, between closely related insect vector species, and even between different populations of the same species [47]. However, little is known about the transcriptome response in insect plant disease vectors due to pathogen invasion. To identify ESTs that play a putative role in the leafhopper immune response, we compared our dataset against >300 *A. gambiae, D. melanogaster,* and *T. castaneum* transcripts with known function in the insect immune response (including several transcript variants of some genes). The 25 *G. nigrifrons* ESTs with highest similarity to known immunity genes of *A. gambiae, D. melanogaster,* and *T. castaneum* are shown in Table 1.

**Table 1:** *G. nigrifrons* ESTs with highest sequence similarity to known immunity genes of *A. gambiae,* and *D. melanogaster*.

*A. gambiae*			*D. melanogaster*		
*Gnig*-EST	E value	Ortholog Immune Function	*Gnig*-EST	E value	Ortholog Immune Function
009993	0	contactin-like putative cell adhesion molecule	005095	0	p38b
019425	0	thioester-containinG protein	002299	0	Pelle associated protein Pellino (Pli)
005039	0	prophenoloxidase	000902	0	Poly-(ADP-ribose) polymerase CG40411-RA, transcript variant A (Parp)
012471	0	signal transducer and activator of transcription	004283	0	Ect4 CG34373-RD, transcript variant D (Ect4), mRNA
004340	0	inhibitor of apoptosis protein	019825	0	Mib1
002299	0	Pellino	002293	0	basket CG5680-RB (bsk)
012767	0	Emi domain; Emilin	010860	0	Helicase 89B CG4261-RA (Hel89B)
008405	0	Trypsin-like serine protease	002299	0	Pellino CG5212-RB, transcript variant B (Pli)
019839	0	Fibronectin type III/ Immunoglobin	003507	0	scribbled CG5462-RD, transcript variant D (scrib)
002849	0	catalase	010867	0	Myosin 61F CG9155-RA, transcript variant A (Myo61F)
018879	0	Peroxidasin	018879	0	Peroxidasin CG12002-RE, transcript variant E (Pxn)
003728	0	scavenger receptor class A-like, C-type lectin	009993	0	Contactin CG1084-RA (Cont)
011660	0	thioester-containing protein	011660	0	Thiolester containing protein II CG7052-RB, transcript variant B (TepII)
012471	0	signal transducer and activator of transcription	004310	0	Hemocyanin
005039	0	prophenoloxidase	002293	0	basket CG5680-RB (bsk)
004340	0	inhibitor of apoptosis protein	004340	0	Bruce CG6303-RA (Bruce)
009993	9.00E-177	contactin-like putative cell adhesion molecule	006257	0	epithelial membrane protein CG2727-RB, transcript variant B (emp),
005039	2.00E-173	prophenoloxidase	019839	0	Neuroglian CG1634-RC, transcript variant C (Nrg)
002271	2.00E-172	scavenger receptor class B	002849	0	Catalase CG6871-RA (Cat)
016562	2.00E-169	START_STARD11-like; Ceramide- binding	005821	0	Ion channel regulatory protein
008405	2.00E-169	Trypsin-like serine protease	010898	0	*olf413*; Copper type II ascorbate-dependent monooxygenase
011660	8.00E-168	thioester-containing protein	019425	0	Macroglobulin complement-related CG7586-RA (Mcr)
012767	1.00E-161	EMI domain; Emilin	010860	1.00E-180	Helicase 89B CG4261-RA (Hel89B),
002849	1.00E-161	catalase	008405	4.00E-180	TequilA CG4821-RD, transcript variant D (Tequila)
002190	7.00E-161	UAS family, FAS-associated factor 1, ubiquitin	005095	5.00E-180	Mpk2 CG5475-RB, transcript variant B (Mpk2)

A total of 194 *G. nigrifrons* ESTs were predicted to be functional in the immune response (Table S4) such as Toll and immune deficiency (IMD) pathways, Jun N-terminal kinase (JNK), and Janus Kinase/signal transducer of activator of transcription (JAK/STAT). In addition, 10 ESTs with the E≤10^−13^, putatively function as pattern recognition proteins (PRPs) including different gram-negative bacteria binding proteins (GNBPs), C-type lectin and scavenger receptors. Of particular interest in this group of PRPs are PGRPs, which are the proteins responsible for sensing and binding of non-self molecules and which activate downstream immune responses. Since the Toll pathway was reported to play an important role in *Drosophila* X virus (DXV) defense [23], ESTs encoding putative key proteins involved in this pathway such as Spaetzle, protein Toll as well as its partners, Pelle and MyD88, Cactus, and Dorsal-related immunity factor (Dif) were identified. Other essential proteins function in IMD pathway such as DREDD and Relish, their corresponding ESTs were also present in *G. nigrifrons*. Although there are eight types of AMPs identified in *D. melanogaster*, there was only one putative AMP gene, defensin, identified in *G. nigrifrons* ESTs. The relatively large percentage of highly similar transcripts is expected, given the high degree of conservation of immune signaling pathways across diverse insect and mammalian taxa [48], [49].

### *G. nigrifrons* ESTs with similarity to RNAi pathway genes

RNAi is a highly conserved gene silencing process triggered by double-stranded RNAs and guided by small interfering RNA (siRNA) that involves post-transcriptional gene silencing (PTGS), which is essential for virus defense and development in insects [50], [51]. Thirty-two Drosophila genes have been implicated in RNAi/PTGS. *G. nigrifrons* ESTs homologous to RNAi/PTGS transcripts were identified using tBLASTx with an (E-value <10^−10^) against nucleotide sequences of *D. melanogaster* RNAi/PTGS genes. Forty-three *G. nigrifrons* ESTs matched to at least one *D. melanogaster* RNAi/PTGS transcript variant, and included 69% of *D. melanogaster* RNAi/PTGS genes (Table 2). Significant matches for some of the *D. melanogaster* RNAi/PTGS genes (e.g. FBgn0262447, FBgn0262432, FBgn0262391) were not identified, likely due to their small size (<100 bp).

**Table 2:** Comparisons of RNAi related genes among *D. melanogaster* and *G. nigrifrons*.

*D. melanogaster* Gene ID	*D. melanogaster* matching transcript ID	Gene Name	*G. nigrifrons* Ortholog Sequence(s)	E-value^b^
FBgn0262739	FBtr0087613	*Argonaute 1*	GnigEST000518	5.00E-89
			GnigEST002602	7.00E-89
			GnigEST006417	1.00E-87
	FBtr0305592		GnigEST009734	0
FBgn0087035	FBtr0308124	*Argonaute 2*	GnigEST001517	5.00E-129
			GnigEST011627	8.00E-129
FBgn0250816	FBtr0299881	*Argonaute 3*	GnigEST010662	0
FBgn0041164	FBtr0073126	*armitage*	GnigEST001564	1.00E-61
			GnigEST004992	1.00E-61
			GnigEST019947	3.00E-124
	FBtr0100641		GnigEST002574	5.00E-24
FBgn0033062	FBtr0086018	*Ars2*	GnigEST000777	0.00E+00
	FBtr0086021		GnigEST001871	0.00E+00
FBgn0041188	FBtr0083100	*Ataxin-2*	GnigEST008087	2.00E-44
FBgn0000146	FBtr0080165	*aubergine*	GnigEST001554	6.00E-69
	FBtr0112793		GnigEST005555	1.00E-92
FBgn0015925	FBtr0089872	*capsuleen*	GnigEST018982	1.00E-115
FBgn0039016	FBtr0084324	*Dicer-1*	GnigEST008045	3.00E-17
			GnigEST009981	0.00E+00
			GnigEST018682	2.00E-155
			GnigEST027158	4.00E-33
			GnigEST027920	1.00E-35
FBgn0034246	FBtr0086904	*Dicer-2*	GnigEST002000	2.00E-162
			GnigEST006630	1.00E-162
FBgn0026722	FBtr0088850	*drosha*	GnigEST002613	2.00E-34
			GnigEST012820	1.00E-44
			GnigEST014952	5.00E-84
			GnigEST032162	2.00E-25
FBgn0028734	FBtr0308123	*Fmr1*	GnigEST000043	2.00E-162
			GnigEST000914	2.00E-162
FBgn0051992	FBtr0100166	*gawky*	GnigEST001451	4.00E-103
			GnigEST002257	1.00E-99
			GnigEST010708	2.00E-133
FBgn0032340	FBtr0080231	*Ge-1*	GnigEST007924	1.00E-86
FBgn0033686	FBtr0087984	*Hen1*	GnigEST009288	2.00E-58
FBgn0032515	FBtr0080497	*loquacious*	GnigEST009073	8.00E-108
FBgn0039861	FBtr0085820	*partner of drosha*	GnigEST012472	3.00E-47
			GnigEST022347	3.00E-80
FBgn0033686	FBtr0087984	*piRNA methyltransferase*	GnigEST009288	2.00E-58
FBgn0004872	FBtr0080166	*piwi*	GnigEST011926	0
FBgn0039168	FBtr0084569	*twin*	GnigEST018227	0
	FBtr0084571		GnigEST032142	6.00E-32

### Validation and expression profiles of *G. nigrifrons* candidate genes

Since reference genes for assaying *G. nigrifrons* gene expression have not been described, we examined expression of six genes previously selected for reference gene validation in other insects using RT-qPCR [52]. Primer pairs for all six genes had a *R* ^2^>0.99 and primer efficiency (*E* values) between 1.8 and 2.2 (Table S5). Stability analysis revealed *RPS13* and *α –TUB* were the most stably expressed genes (Figure S2), and *RPS13* was chosen based on comparisons with other insects [53].

**Figure 2:**
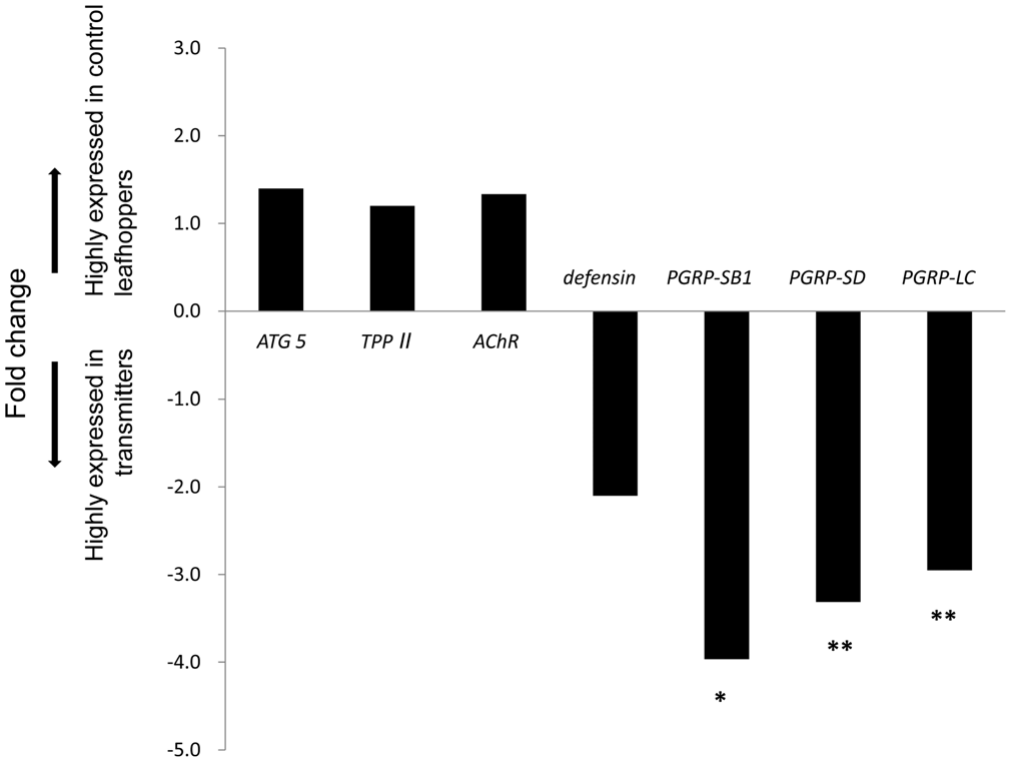
Expression profiles of genes involved in *G. nigrifrons* immune response detected by RT-qPCR. Each bar represents the fold change of *G. nigrifrons* transmitters versus control leafhoppers. The gene expression level was nomalized to *ribosomal protein S13* (*RPS13*), and the expression level of control leafhoppers was used as calibrator to calculate the fold change. Fold change was calculated based on a mean of three biological replicates concluding 30 individuals. Bars above the X axis show the genes expressed slightly higher in transmitters, while the bars below are genes down regulated in transmitters. *P<0.05; **P<0.01.

Gene expression patterns for seven selected genes implicated in insect innate immunity were tested using RT-qPCR, including three *PGRP*s (two representing short class and one representing long class), as well as *AChR*, *ATG 5*, *TPP-II* and *defensin* (the only AMP EST in our database) [19], [28], [54]. RNAs were isolated from *G. nigrifrons* individuals that were either experimentally determined to be MFSV transmitters or control leafhoppers. RNA used for RT-qPCR had an A_260_/A_280_ between 1.8 and 2, and the PCR efficiencies for the candidate gene primers were between 1.8 and 2.2 (Table S1). The fold difference in expression for transmitters versus control leafhoppers ranged from −4.0 to 1.4 (Figure 2). No significant differences in *ATG5, AChR, TPP-II* or *defensin* expression were detected. However, expression of the *PGRP* genes was significantly lower in transmitters compared to control leafhoppers.

Previous studies suggested roles for *ATG5, AChR, TPP-II* or *defensin* in the innate immune response. A role for autophagy in controlling vesicular stomatitis virus (VSV) replication was indicated in *D. melanogaster* [55]. TPP-II, a multi-function peptidase, was reported to be involved in protein turnover and immune response through processing antigen epitopes [56], [57]. AChR was suggested as a receptor for rabies virus (Rhabdoviridae) [58]. *Defensin* is induced during bacterial infection after activation of the Toll pathway by the recognition and binding of PAMPs to PGRP-SA or SD [48], [59], [60]. However no significant expression difference between transmitters and control leafhoppers was detected for any of these four genes. A similar lack of an effect of MMV infection was found in *P. maidis* for *ATG3* and *TPP-II* [28], and for *defensin* in Sigma virus (SIGMAV)-infected and non-infected *Drosophila* [19]. Therefore, although these genes have roles in innate insect immunity, their expression does not seem to respond to long-term rhabdovirus infection in planthoppers or leafhoppers.

Recent studies have indicated a role of *PGRP*s in insect virus defense [19], and our data shows a potential interaction between putative *G. nigrifrons PGRPs* and rhabdovirus infection. All three putative-*PGRPs* tested (*PGRP-SB1*, *PGRP-SD* and *PGRP-LC*) showed significantly lower expression in MFSV transmitters compared to control leafhoppers, and the fold changes ranged from −2.9 to −4.0 (Figure 2). Pathogens, such as bacteria and fungi, are recognized by PGRPs, which subsequently trigger downstream AMPs production through Toll or IMD immune pathway [22]. In *Drosophila*, infection with the rhabdovirus SIGMAV also alters expression of *PGRP-SB1* and *PGRP-SD*; however in this case, increased transcript levels were found with these genes in infected *Drosopila* compared to un-infected [19], [23]. Several non-mutually exclusive factors may explain the different expression profiles of these three putative *PGRPs* in the *G. nigrifrons*/MFSV and *D. melanogaste*/SIGMAV systems. SIGMAV are known insect pathogens, whereas the pathogenicity of MFSV in *G. nigrifrons* has not been determined. For example, SIGMAV, which is also a member of *Rhabdoviridae*, is a natural viral insect pathogen widespread in *D. melanogaster* populations, and is vertically transmitted [61]. Alternatively, MFSV is transmitted only through feeding on infected plant hosts; vertical transmission has not been observed in *G. nigrifrons* [7], [11]. The difference in virus acquisition and pathogenicity may elicit different immune responses.

Additionally, we hypothesize that MFSV might actively manipulate vector immune response in terms of reduced expression of these three *PGRPs* in order to successfully replicate within *G. nigrifrons* transmitters and acquirers. Virus manipulation has been observed with the *Togavirus* Semliki Forest Virus (SFV) within infected cell lines of the mosquito *Aedes albopictus*; in this case, activation of immune defense pathways was suppressed [62]. Manipulation may be through direct interactions with the insect vector, or by altering physiological processes of the plant hosts such that an elevated plant defense response impacts insect defenses which consequently favors virus replication. Although this hypothesis is untested with *G. nigrifrons*-MFSV, further work aimed at describing the expression profiles of other *PGRPs* among all three *G. nigrifrons* classes (transmitters, acquirers, non-acquirers) will help understand the interactions between PGRPs and MFSV proteins that lead to virus transmission.

Our study represents the first transcriptome characterization of a leafhopper species, which includes 38,240 transcripts. Significant sequence similarity existed in immune defense genes existed between *G. nigrifrons* and other well-characterized insects. Additionally, we have identified several putative components of the RNAi pathway, which will enable future development of functional evaluation of the role of this important pathway in transmission of MFSV. The down-regulation of *PGRPs* in MFSV transmitters suggests a possible interaction with rhabdovirus transmission by vectors, although additional research is required to define the mechanism. The results presented expand molecular characterization of plant virus vectors and will help further understand the mechanisms of plant virus vector competence.

## Supporting Information

Figure S1 **Gene ontology (GO) terms for** ***G. nigrifrons*** **EST (contigs and singletons).** The pie charts were generated based on A. Biological process; B. Molecular function; C. Cellular component.
(PDF)

Figure S2 **Comparison of reference genes for** ***G. nigrifrons*** **using geNorm.** Genes with lower average expression stability *M* are more stable among all treatments. *α-TUB*, alpha tubulin; *EF-1α*, elongation factor 1-alpha; *GAPDH*, glyceraldehyde-3-phosphate dehydrogenase; *SDHA*, succinate dehydrogenase; *RPL3*, ribosomal protein L3; *RPS13*, ribosomal protein S13.
(PDF)

Table S1 **Primer sequences and efficiencies of candidate genes evaluated for differential expression among MFSV transmitters and control** ***G. nigrifrons*** **(RT-qPCR).**
(DOCX)

Table S2 ***G. nigrifrons*** **ESTs that have the most significant alignment (E-value <10^−180^) with genes in the Swiss-Prot database.**
(XLSX)

Table S3 **Results from KEGG pathway analysis.**
(XLSX)

Table S4 **A total of 194** ***G. nigrifrons*** **ESTs were predicted to be implicated in immune response.**
(XLSX)

Table S5 **Primer sequences, efficiencies and correlation of potential RT-qPCR reference genes for** ***G. nigrifrons.***
(DOCX)
