# Supplementary figures and images for: Transcriptome of the Plant Virus Vector Graminella nigrifrons, and the Molecular Interactions of Maize fine streak rhabdovirus Transmission
Source: PLoS One. 2012 Jul 12;7(7):e40613. doi: 10.1371/journal.pone.0040613 (PMC3395673; doi:10.1371/journal.pone.0040613)

Supplementary figure S1.

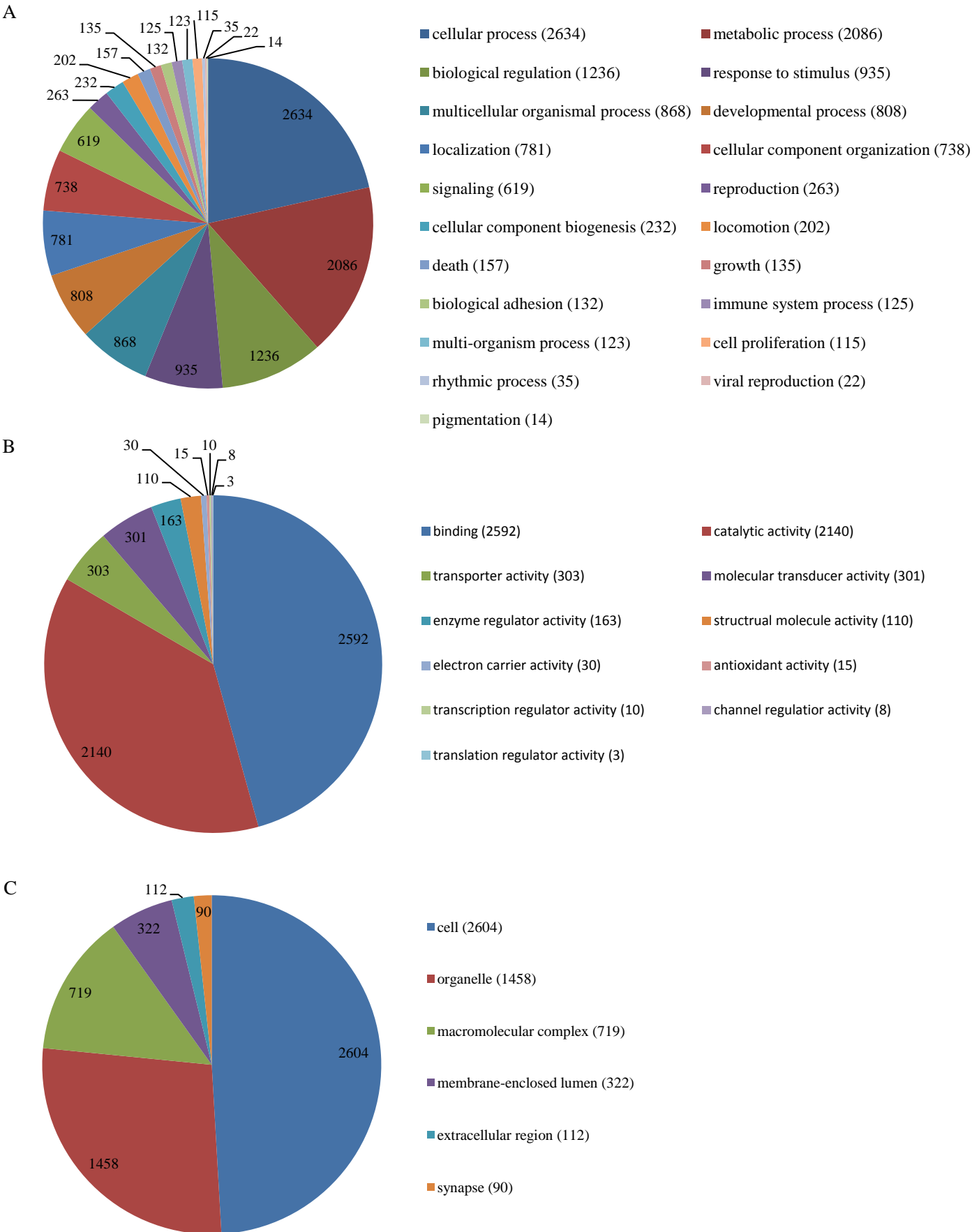

Supplement: Figure S1 — Gene ontology (GO) terms for G. nigrifrons EST (contigs and singletons). The pie charts were generated based on A. Biological process; B. Molecular function; C. Cellular component. (PDF) [file pone.0040613.s001.pdf]

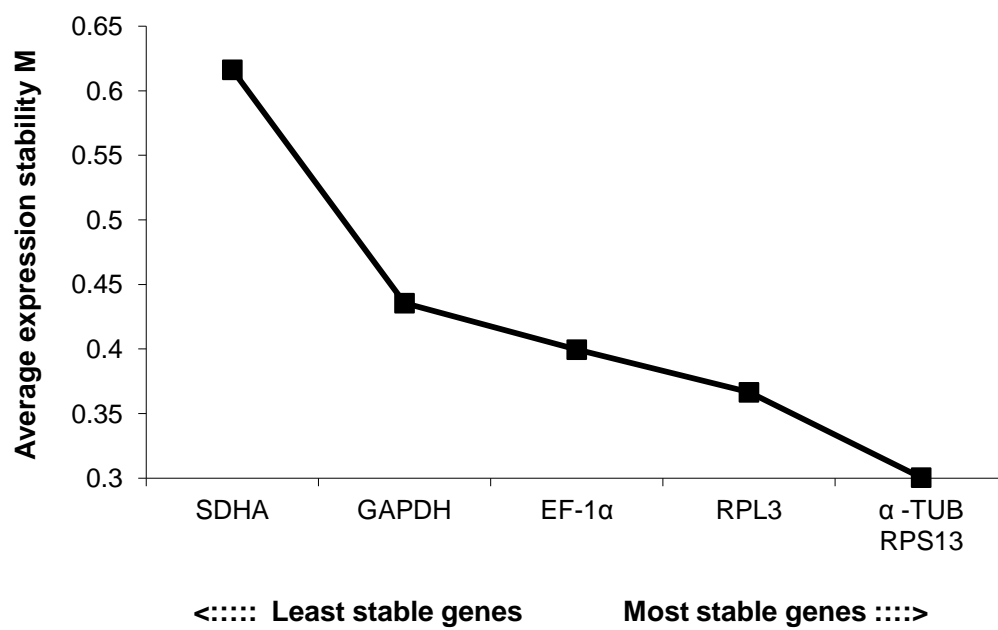

Supplement: Figure S2 — Comparison of reference genes for G. nigrifrons using geNorm. Genes with lower average expression stability M are more stable among all treatments. α-TUB, alpha tubulin; EF-1α, elongation factor 1-alpha; GAPDH, glyceraldehyde-3-phosphate dehydrogenase; SDHA, succinate dehydrogenase; RPL3, ribosomal protein L3; RPS13, ribosomal protein S13. (PDF) [file pone.0040613.s002.pdf]
