# Supplementary material for: Transcriptome of the Plant Virus Vector Graminella nigrifrons, and the Molecular Interactions of Maize fine streak rhabdovirus Transmission
Source: PLoS One. 2012 Jul 12;7(7):e40613. doi: 10.1371/journal.pone.0040613 (PMC3395673; doi:10.1371/journal.pone.0040613)
Supplement: Table S1 — Primer sequences and efficiencies of candidate genes evaluated for differential expression among MFSV transmitters and control G. nigrifrons (RT-qPCR). (DOCX) [file pone.0040613.s003.docx]

Supplementary Table 1.

| Name^a^ | Primer Sequence | Length (bp) | Tm (°C) | Efficiency^b^ |
| --- | --- | --- | --- | --- |
| AChR-F | 5’- CGACAGCATCAGCGACAT -3’ | 18 | 55.4 | 2.10 |
| AChR-R | 5’- ATGTGGTGTTGTATGAATGTTACG -3’ | 24 | 53.6 | 2.10 |
| ATG5-F | 5’- CTACCGTGGAACATTACC -3’ | 18 | 49.8 | 1.84 |
| ATG5-R | 5’- TGACACAAGACATGAAGTAT -3’ | 20 | 48.7 | 1.84 |
| Defensin-F | 5’- GTCTCCTATTGGCTACAG -3’ | 18 | 48.9 | 1.96 |
| Defensin-R | 5’- GCATACGAACACCTAATC -3’ | 18 | 47.6 | 1.96 |
| PGRP-SB1-F | 5’- ACTAACTCAAGACAGACATAT -3’ | 21 | 48.1 | 1.83 |
| PGRP-SB1-R | 5’- CTCTCGTCCTTCATACAC -3’ | 18 | 49.2 | 1.83 |
| PGRP-SD-F | 5’- TATACGAAGGAAGAGGTT -3’ | 18 | 46.6 | 1.86 |
| PGRP-SD-R | 5’- GCCAATGTAAGCAATATC -3’ | 18 | 45.8 | 1.86 |
| PGRP-LC-F | 5’- CGTCTTCTTCACCTACACAA -3’ | 20 | 52.0 | 2.12 |
| PGRP-LC-R | 5’- TGCCTCCTGCTGTATGTT -3’ | 18 | 53.7 | 2.12 |
| TPP II-F | 5’- ACCTCTTCCTTATTGCCTAA -3’ | 20 | 50.6 | 2.09 |
| TPP II-R | 5’- ACAGTTCCTCTTCCATCAT -3’ | 19 | 50.5 | 2.09 |

^a^AChR, acetylcholine receptor subunit alpha-L1; ATG 5, autophagy protein 5; PGRP, peptidoglycan recognition protein; TPP II, tripeptidyl peptidase II

^b^Efficiency was calculated by the equation *E* = 10^[-1/slope]^.
