# Supplementary material for: Transcriptome of the Plant Virus Vector Graminella nigrifrons, and the Molecular Interactions of Maize fine streak rhabdovirus Transmission
Source: PLoS One. 2012 Jul 12;7(7):e40613. doi: 10.1371/journal.pone.0040613 (PMC3395673; doi:10.1371/journal.pone.0040613)
Supplement: Table S5 — Primer sequences, efficiencies and correlation of potential RT-qPCR reference genes for G. nigrifrons. (DOCX) [file pone.0040613.s007.docx]

Supplementary Table 5.

| Name ^a^ | Primer Sequence | Length (bp) | Tm (°C) | *E* ^b^ | *R*^2 c^ |
| --- | --- | --- | --- | --- | --- |
| *α-TUB* | 5’- GGGAAAGGAAGCCTAGTTGG -3’ | 20 | 55.4 | \| 1.90 \| \| --- \| | 0.998 |
|  | 5’- TTGTTTTCCGAGACCAGTCC -3’ | 20 | 54.9 |  |  |
| *EF-1α* | 5’- CTACACACCCGTCCTCGATT -3’ | 20 | 56.5 | 1.99 | 0.999 |
|  | 5’- ACTTGGGGTTGTCCTCAGTG -3’ | 20 | 57.1 |  |  |
| *GAPDH* | 5’- CGATGTTCGTTGTTGGTGTC -3’ | 20 | 54.3 | 2.01 | 0.997 |
|  | 5’- CATCAGTCCCTCCACGATCT -3’ | 20 | 56.1 |  |  |
| *SDHA* | 5’- TCTACCGATGGAACCCTGAC -3’ | 20 | 56.0 | 1.95 | 0.994 |
|  | 5’- TTGATCAGAGCATCCAGCAC -3’ | 20 | 55.1 |  |  |
| *RPL3* | 5’- CTAAGGCGTCCAAGAAGTGG -3’ | 20 | 55.5 | 1.96 | 0.999 |
|  | 5’- CTTCATCTGGGTGTGAGCAA -3’ | 20 | 55.0 |  |  |
| *RPS13* | 5’- TCCCAGTCTGCTCTTCCCTA -3’ | 20 | 57.1 | 1.99 | 0.996 |
|  | 5’- CACCGTGAGAGTCCCTCAAT -3’ | 20 | 56.5 |  |  |

^a^*α-TUB*, alpha tubulin, *EF-1α*, elongation factor 1-alpha, *GAPDH*, glyceraldehyde-3-phosphate dehydrogenase, *SDHA*, succinate dehydrogenase, *RPL3*, ribosomal protein L3, *RPS13*, ribosomal protein S13.

^b^*E*, PCR efficiency was calculated by the equation *E* = 10^[-1/slope]^.

^c^*R*^2^, correlation co-efficient was calculated from standard curve.
